# Who Starts? Factors Associated with Starting Antiretroviral Therapy among Eligible Patients in Two, Public HIV Clinics in Lilongwe, Malawi

**DOI:** 10.1371/journal.pone.0050871

**Published:** 2012-11-30

**Authors:** Caryl Feldacker, Derek Johnson, Mina Hosseinipour, Sam Phiri, Hannock Tweya

**Affiliations:** 1 Lighthouse Trust, Lilongwe, Malawi; 2 International Training and Education Center for Health (I-TECH), University of Washington, Seattle, Washington, United States of America; 3 University of North Carolina Project, Lilongwe, Malawi; 4 University of North Carolina School of Medicine, Chapel Hill, North Carolina, United States of America; University of New South Wales, Australia

## Abstract

**Background:**

Lighthouse Trust operates two, public, integrated HIV clinics, Lighthouse (LH) and Martin Preuss Center (MPC), in Lilongwe, Malawi. Approximately 20% of patients eligible for antiretroviral therapy (ART) do not start ART. We explore individual and geographic factors that influence whether ART-eligible patients initiate ART.

**Methods:**

Adult patients eligible for ART between 2008–2011 were included. Analysis was stratified by clinic. Using logistic regression, we evaluated factors associated with initiating ART including gender, age, body mass index (BMI), employment, tuberculosis (TB), eligible at initial registration, WHO stage, CD4, months in pre-ART care (from initial registration to eligibility date), and patient neighborhood distance to clinic.

**Results:**

Of 14,216 study patients, 4841 were from LH; 9285 were from MPC. At LH and MPC, respectively, median age was 34.2 and 33.8 years; median BMI was 22.0 and 20.6; and median distance was 5.6 and 4.9 Km. In multivariate models, odds of starting ART was highest among those older than 35 years and those eligible for ART based on WHO stages 3–4 vs. those in WHO stages 1–2 with CD4<250. Patients with 1–12 months in pre-ART were at least 11 times more likely to start ART than peers with less pre-ART time. At LH, living 2.5–5 Km from the clinic increased the likelihood of starting ART over patients living closer.

**Conclusions:**

Length of the pre-ART period is the most significant predictor of starting ART among eligible patients. Better understanding of motivation for retention in pre-ART care may reduce attrition along the treatment cascade.

## Introduction

As access to life-sustaining antiretroviral therapy (ART) increases throughout much of sub-Saharan Africa, attention is needed to improve quality services throughout the continuum of HIV-related care, including the period before ART initiation. Previous studies from sub-Saharan Africa examine loss to follow up (LTFU) among HIV-infected patients who were not yet eligible to start ART [1], [2], [3], [4] and factors that influence LTFU after ART initiation [5], [6], [7], [8], [9], [10]. However, the period between ART eligibility and ART initiation, during pre-ART care, is a time of high vulnerability [11], [12]. In sub-Saharan Africa, pre-ART program attrition or discontinuation of care for any reason including LTFU and death, ranges from 14%–84% [13], illustrating a sizeable gap in the continuum of ART services. Patients who are lost to attrition after becoming eligible may eventually enter care later and sicker [14], [15], [16], decreasing the overall effectiveness, and increasing the costs, of HIV treatment programs [13]. Hence, greater understanding of the drivers of attrition during this critical stage would strengthen efforts to retain patients during this transition.

In Malawi, program attrition across the continuum of HIV-related care is of concern. By 2011, the Ministry of Health reported national ART program coverage at 67% [17], indicating both great progress in ART scale up as well as lingering weaknesses moving ART-eligible patients into life-long treatment. National adult HIV prevalence remains high at approximately 12%; the majority of HIV-infected persons seek HIV-related care at public clinics [17]. Lighthouse Trust, the largest provider of HIV-related services including ART in Lilongwe, Malawi and a flagship public provider of HIV-related services [18], runs two high-burden, integrated ART clinics: Lighthouse clinic (LH) on the campus of Kamuzu Central Hospital and the Martin Preuss Center (MPC) on the campus of Bwaila Hospital. Although national-level pre-ART attrition is unknown, approximately 20% of eligible patients do not start ART at LH and MPC, likely indicative of a similarly sized gap in the continuum of care at other public clinics.

In this study we explore the individual and geographic factors that influence whether ART-eligible patients at LH and MPC initiate ART. Increasing knowledge of the drivers of, and impediments to, ART initiation among eligible patients will help inform programmatic and policy efforts to reduce attrition during this critical period of entry to ART care.

## Methods

### Ethics Approval

According to the Malawi National Health Science Research Committee (NHSRC) guidelines, retrospective reviews of existing programmatic data are exempt from formal consenting procedures. Lighthouse received approval from the NHSRC for this study in 2011 for analysis of routine programmatic data and publication of results from operations research.

### Study Setting

The Lighthouse Trust partners with both the Malawi Ministry of Health (MoH) and the District Health Office (DHO) in Lilongwe to operate two integrated HIV clinics. Lighthouse (LH) Clinic opened in 2001. The Martin Preuss Center (MPC), located near the central bus station, opened in Dec 2006 as a purposeful TB/ART integration site [19]. Combining patients at both clinics, the Lighthouse Trust is the largest public provider of free ART in the Central Region with over 22,000 patients in HIV-related care. From 2008 onwards, both LH and MPC used a real-time, touchscreen-based electronic data system (EDS) for patient management which improved clinic performance and reduced data errors [20]. The EDS collects HIV testing details, residential location, demographics, and World Health Organization (WHO) stage at registration. The EDS also includes CD4 test results, opportunistic infections, reported side effects, and drug dispensing records. Pre-ART patient information is not stored longitudinally in the EDS; only registration, demographic, and most recent eligibility-relevant information is retained.

Although the MoH revised national ART guidelines in June, 2011 [21], Malawi’s 2008 National ART guidelines used at Lighthouse and all ART clinics defined ART eligibility for adults as meeting one of three criteria: 1) WHO stage 1 or 2 in combination with CD4 cell count of less than 250 cell/mm^3^; 2) WHO stage 3; or 3) WHO stage 4 [22]. Pre-ART patients, HIV-infected individuals who have not started ART, are reviewed every 2 months to provide co-trimoxazole therapy (CPT) and to evaluate for any potential stage 3 or 4 defining conditions. Pre-ART patients should also undergo routine CD4 testing at 6-month intervals to monitor for ART eligibility; however, weaknesses in the laboratory system and problems with CD4 machines may delay or prevent CD4 testing or results provision. Also, patients who have an infection or condition indicative of a WHO 3 or WHO 4 condition, including TB, are immediately eligible for ART and may not have a CD4 test ordered or results recorded. Pre-ART patients may seek care between visits if they are sick.

Clinical procedures and hours of operation (Monday to Friday 7∶30am−4∶30pm, Saturday from 8am to 12pm) are identical at MPC and LH. At the first clinic visit and all subsequent pre-ART visits, patients are clinically staged and those with Stage 3 or 4 conditions are immediately informed of their ART eligibility by a clinician. Those who are stage 1 or 2 typically undergo CD4 testing to determine eligibility and are scheduled to return in 2 weeks for the result. Before an individual can begin ART, they are required to attend an ART education session with a guardian, usually a family member or friend. Eligible patients may start ART on the same day as the initial clinic visit or on the day of receiving their CD4 result if education and guardian requirements are met. Pregnant women are eligible for expedited start procedures [23] and were, therefore, excluded. Transfer patients were excluded due to incomplete data on eligibility criteria or pre-ART care prior to transfer.

### Independent Variables and Covariate Definitions

Adult patients (>15 years) included in the study were informed of their eligibility for ART during a clinic visit between January 1, 2008–June 30, 2011. WHO stage 3 and 4 patients would be eligible and informed at a single visit; patients eligible by CD4 would require an additional visit before being informed. The binary outcome of interest was ART start or not as defined by the date of first ART drug dispensation. ART start dates were included through the study end date of September 30, 2011. Year of enrolment was categorized into 5 groups: 2007 or earlier; 2008; 2009; 2010; or from January 1 through June 30, 2011. Additional independent demographic variables included: sex; age at registration classified into 10 year age categories; body mass index (BMI) within 30 days of eligibility dichotomized as less than or equal to 18.5 or higher; and self-reported occupation classified as any current work vs. unemployment, including student or housewife status. Healthcare service related variables included ART eligibility at initial registration and diagnosis of TB at eligibility. Time in the pre-ART program was defined as months from initial clinic registration to the date a patient was informed of eligibility.

For location information, Euclidean distance in kilometers (Km) between LH or MPC clinic and patient village or Area locations was determined using Google Earth [24]. Location information was available for 8447 MPC (91%) and 4138 LH (85%) patients.

### Statistical Analysis

Analysis was stratified by clinic, LH or MPC. Chi-square tests determined significant differences between eligible patients who started and those who did not, by clinic. Logistic regression models assessed factors associated with starting ART in univariate and multivariate models. T-tests determined significance of individual variables; alpha was set to 0.05. Age and gender were included in multivariate models *a priori*. Otherwise, significant factors at the p<0.05 level in univariate models were included in multivariate models. TB at eligibility, ART eligible at 1^st^ visit, and CD4 cell count were not included in multivariate models because of their correlation with eligibility criteria. Analysis was conducted using STATA 11.0 [25].

## Results

**Figure 1:**
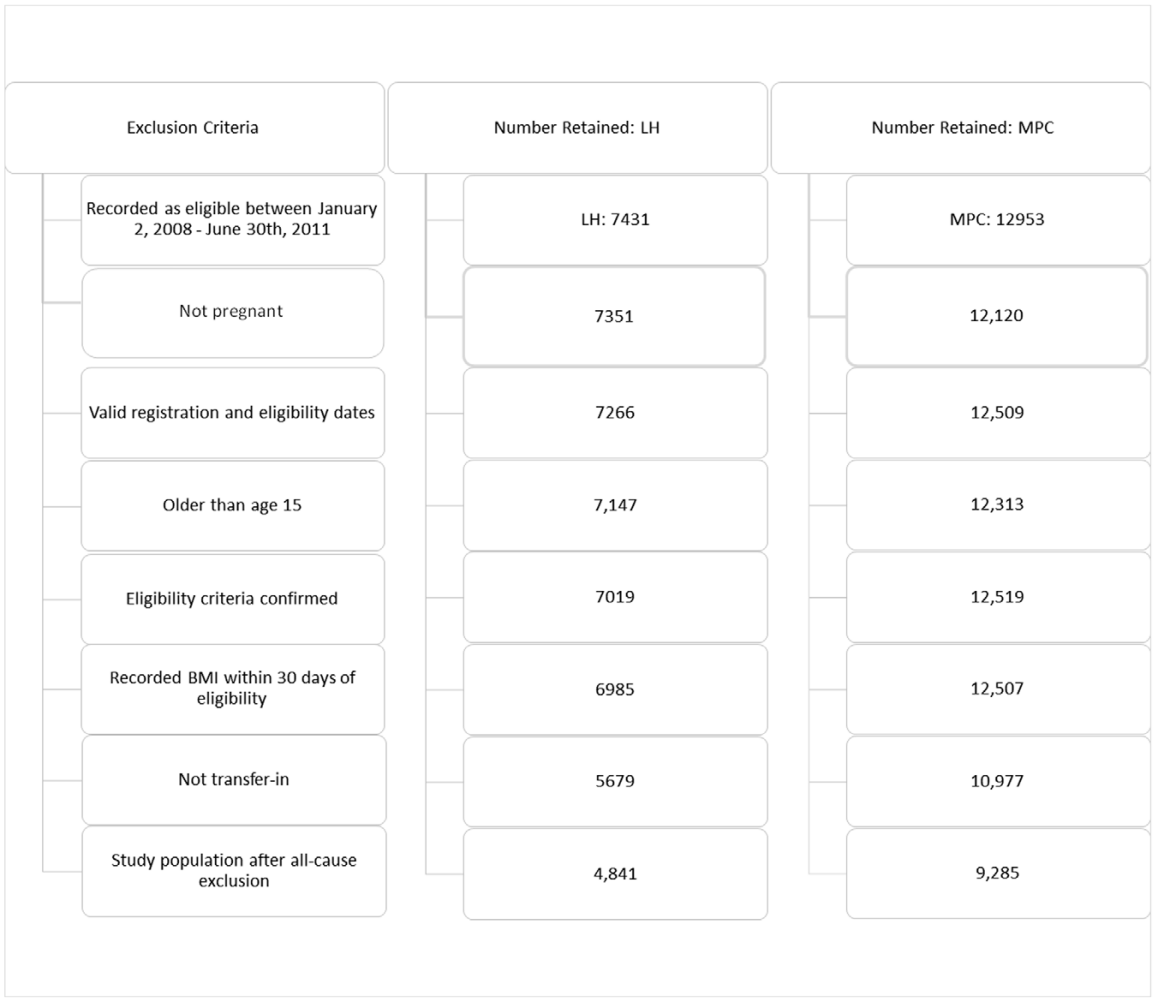
Flow chart of patient population characteristics retained in the study.

At LH and MPC, respectively, 7 431 and 12 953 patients were presumed eligible for ART between January 1, 2008 and June 30^th^, 2011; 4 841 LH and 9 285 MPC patients were included in the study after removing pregnant women, transfers and other patients with incomplete or inaccurate records (Figure 1). At LH, 3 929 (81.2%) of 4 841 eligible patients started ART before September 30, 2011. Of 9 285 MPC patients, 7 212 (77.7%) started ART during the study period. Baseline characteristics of LH and MPC study patients were similar except MPC included a larger proportion with tuberculosis and patients eligible for ART at first visit (Table 1).

Table 2 describes differences in those who start ART and not, by clinic. At both clinics, those under 24 years of age, eligible for ART by CD4 criteria <250 cells, and without TB at baseline were less likely to begin ART. Patients who did not start ART as compared to those who started ART were more likely to be eligible at first visit (83.7% vs. 38.9% at LH and 92.1% vs. 53.3% at MPC) and have less than 1 month in the pre-ART period (85.2% vs 45.3% at LH and 93.8% vs. 57.2% at MPC). The proportion of eligible patients who initiated ART improved over time at MPC but decreased or remained stable at LH.

**Table 1:** Descriptive statistics of all eligible patients, by clinic.

	Lighthouse		MPC	
	N = 4841*	%	N = 9285*	%
Started ART				
Yes		81.2		77.6
No		18.8		22.4
Sex				
Female		48.7		48.0
Male		51.3		52.0
Age				
Mean (SD)		35.5 (9.3)		35.2 (9.4)
Median (IQR)		34.2 (29.0–40.5)		33.8 (28.5–40.2)
Age group				
15–24		10.1		10.8
25–34		43.5		45.1
35–44		31.7		29.3
≥45		14.7		14.8
Employment				
Any		70.1		78.6
None		29.9		21.4
BMI (Kg/h^2^)				
Mean (SD)		22.6 (4.0)		21.0 (3.5)
Median (IQR)		22.0 (20.0–24.4)		20.6 (18.8–22.7)
CD4 count (cell/mm^3^)	2328		2931	
Mean (SD)		155.8 (102.2)		150.0 (88.9)
Median (IQR)		158 (84–217)		150 (87–207)
WHO stage				
1&2		43.1		34.9
3		37.8		52.5
4		19.0		12.6
TB diagnosed at eligibility***				
Yes		4.7		24.4
No		95.3		75.6
ART eligible at first visit				
Yes		47.3		61.9
No		52.7		38.1
Months in pre-ART				
Mean (SD)		13.1 (32.4)		3.88 (12.9)
Median (IQR)		0.42 (0–3.6)		0 (0–2.3)
Registration year				
≤2007		13.5		3.1
2008		22.5		30.3
2009		27.1		28.1
2010		24.9		28.1
2011**		12.0		10.4
Year of eligibility				
2008		28.1		29.8
2009		29.2		28.0
2010		28.0		29.6
2011**		14.7		12.7
Distance from clinic (Km)	4138		8447	
Mean (SD)		8.9 (13.8)		7.4 (13.2)
Median (Range)		5.6 (4.0–9.6)		4.9 (3.9–6.5)

* unless otherwise specified;

** Through June 30, 2011;

*** Included in WHO 3 or WHO 4.

Univariate and multivariate models, stratified by clinic, are presented in Table 3 (columns 2, 3 for LH, columns 4, 5 for MPC). At both clinics, age plays a significant role. Patients ages 15–24 were less likely at LH (AOR 0.70; CI 0.53–0.93) and MPC (AOR 0.70; CI: 0. 0.59–0.84) to start ART while older patients, 35–45 years, were more likely to start at both LH (AOR 1.41; CI: 1.16–1.77) and MPC (AOR 1.22; CI: 1.06–1.40) than those ages 25–34 years. At both clinics, eligibility criteria were significantly associated with starting ART. Both LH patients in WHO stage 3 (AOR 5.14; CI: 4.12–6.36) or WHO stage 4 (AOR 4.99; CI: 3.82–6.51) and MPC patients in WHO stage 3 (AOR 14.1; CI: 11.9–16.6) or WHO stage 4 (AOR 11.9; CI: 973–14.8) were more likely to start than those eligible by CD4. At both clinics, time in pre-ART dramatically increased the odds of starting ART. Patients with 1–3 months in pre-ART were more than 28 (AOR 28.9; CI: 19.29–43.52) and 49 times (AOR 49.0; CI: 36.9–65.2) more likely at LH and MPC, respectively, to start ART than patients with less than one month in the pre-ART period. Patients with a year or more time in the pre-ART period at LH (AOR 12.69; CI: 7.15–23.0) and MPC (AOR 63.2; CI: 36.4–115.2) were also more likely to start ART than their peers with little pre-ART time. Distance was only included in the multivariate model at LH. LH patients who lived between 2.5–5 km (AOR 1.54; CI 1.12–2.16) were more likely to start ART than patients who lived closer.

**Table 2:** Comparison of ART eligible patients who started and did not start ART, by clinic.

	Lighthouse			MPC		
	No Start (%)(N = 912)	Started ART (%) (N = 3,929)	p-value	No Start (%)(N = 2,073)	Started ART (%) (N = 7,212)	p-value
Sex			0.81			0.00
Female	51.6	51.2		55.8	50.9	
Male	48.4	48.8		44.2	49.1	
Age group			0.00			0.00
15–24	14.9	8.9		15.9	9.3	
25–34	47.2	42.7		47.1	44.5	
35–44	25.8	33.1		24.8	30.6	
≥45	12.2	15.3		12.2	15.6	
Employment			0.00			0.00
Any	75.1	68.9		76.0	79.4	
None	24.9	31.1		23.9	20.6	
BMI (Kg/h^2^)			0.01			0.03
<18.5	9.0	11.9		19.9	22.1	
≥18.5	91.0	88.1		80.1	77.9	
Eligibility criteria			0.00			0.00
CD4≤250+ Stage 1&2	55.8	40.4		49.2	30.8	
WHO Stage 3	28.3	39.9		39.8	56.2	
WHO Stage 4	15.9	19.7		11.0	13.1	
TB diagnosis at eligibility*			0.01			0.00
Yes	2.9	5.1		10.6	28.3	
No	97.1	94.9		89.4	71.7	
ART eligible at first visit			0.00			0.00
Yes	83.7	38.9		92.1	53.3	
No	16.3	61.1		7.9	46.7	
Months in pre-ART			0.00			0.00
0–1	85.2	45.3		93.8	57.2	
>1–3	3.5	25.6		3.3	20.0	
>3–6	1.1	5.9		0.8	8.6	
>6–12	1.0	4.1		0.6	4.4	
≥12	9.3	20.2		1.5	9.8	
Year of registration□			0.00			0.00
≤2007	10.4	89.6		3.8	96.2	
2008	19.7	80.3		26.6	73.4	
2009	21.6	78.4		24.5	75.5	
2010	17.9	82.1		20.6	79.4	
2011Þ	22.3	77.7		14.3	85.7	
Year of eligibility□			0.05			0.00
2008	17.9	82.1		26.8	73.2	
2009	21.2	78.8		24.9	75.1	
2010	17.4	82.6		19.5	80.5	
2011Þ	18.6	81.4		12.9	87.1	
Distance from clinic (Km)			0.12			0.04
<10 km	81.5	78.9		87.1	85.1	
≥10 km	18.5	21.0		12.9	14.9	
Distance from clinic (Km)			0.02			0.49
0–2.49	12.3	8.7		9.4	9.1	
2.5–4.99	17.7	20.5		42.9	42.0	
5–9.99	51.5	49.7		34.7	34.1	
10–49.99	17.6	19.6		12.4	14.2	
50–100	0.5	0.8		0.2	0.3	
>100	0.4	0.6		0.4	0.4	

Results from chi-square tests of associations within clinic.

Þ Through June 30, 2011;

□ row percentages.

* Included in WHO 3 or WHO 4.

**Table 3:** Results from univariate and multivariate logistic regression models assessing risk factors associated with starting ART vs. not starting ART among eligible patients, stratified by clinic.

	Lighthouse		MPC	
	OR	AOR[n = 4138]	OR	AOR[n = 9285]
Sex				
Male	1.00	1.00	1.00	1.00
Female	0.98(0.79–1.13)	1.21(0.98–1.45)	0.82***(0.74–0.91)	0.96(0.83–1.10)
Age group				
15–24	0.67***(0.54–0.84)	0.70*(0.53–0.93)	0.61***(0.53–0.71)	0.70***(0.59–0.84)
25–34	1.00	1.00	1.00	1.00
35–45	1.43***(1.20–1.69)	1.41***(1.16–1.77)	1.31***(1.16–1.47)	1.22**(1.06–1.40)
≥45	1.38**(1.10–1.74)	1.56**(1.16–2.07)	1.35***(1.16–1.5)	1.34***(1.12–1.61)
Employment				
None	1.00	1.00	1.00	1.00
Any	0.73***(0.62–0.86)	0.87(0.67–1.10)	1.22**(1.08–1.36)	0.89(0.76–1.05)
BMI (Kg/h^2^)				
<18.5	1.00	1.00	1.00	1.00
≥18.5	0.73*(0.57–0.94)	0.85(0.63–1.16)	0.87*(0.77–0.98)	1.04(0.90–1.20)
Eligibility criteria				
CD4≤250+ WHOStage 1&2	1.00	1.00	1.00	1.00
WHO Stage 3	1.93***(1.64–2.28)	5.14***(4.12–6.36)	2.25***(2.02–2.49)	14.1***(11.9–16.6)
WHO Stage 4	1.69***(1.38–2.07)	4.99***(3.82–6.51)	1.89***(1.61–2.22)	11.9***(973–14.8)
TB at eligibility				
No	1.00		1.00	
Yes	1.84**(1.21–2.78)		3.32***(2.86–3.86)	
ART eligible at first visit				
No	1.00		1.00	
Yes	0.12***(0.10–0.15)		0.10***(0.08–0.12)	
Months in pre-ART				
0–1	1.00	1.00	1.00	1.00
>1–3	14.11***(9.72–20.49)	28.9***(19.29–43.52)	10.15***(7.90–13.05)	49.0***(36.9–65.2)
>3–6	10.02***(5.29–18.9)	23.34***(11.26–48.40)	17.3***(10.6–28.1)	98.6***(59.3–163.9)
>6–12	7.63***(3.88–15.02)	11.5***(5.64–23.34)	12.4***(6.9–22.1)	60.2***(32.5–111.5)
≥12	4.06***(3.20–5.15)	12.69***(7.15–23.00)	10.7***(7.44–15.4)	63.2***(36.4–115.2)
Year of registration				
≤2007	1.00	1.00	1.00	1.00
2008	0.47***(0.35–0.63)	1.95*(1.05–3.62)	0.11***(0.06–0.20)	1.00(0.48–2.10)
2009	0.42***(0.32–0.56)	2.1*(1.03–4.51)	0.12***(0.07–0.22)	1.210.45–3.24)
2010	0.53***(0.40–0.71)	2.130.80–5.63)	0.15***(0.08–0.28)	0.910.24–3.37)
2011Þ	0.40***(0.29–0.55)	0.56(0.12–2.61)	0.23***(0.12–0.44)	2.170.46–10.21)
Year of eligibility				
2008	1.00	1.00	1.00	1.00
2009	0.81*(0.67–0.97)	0.79(0.47–1.31)	1.11(0.98–1.25)	0.94(0.47–1.82)
2010	1.04(0.85–1.26)	1.01(0.52–1.98)	1.52***(1.34–1.73)	1.74(0.65–4.64)
2011Þ	0.95(0.75–1.20)	3.80(0.99–14.1)	2.49***(2.06–3.01)	0.86(0.25–2.9)
Distance from clinic (Km)	[n = 4138]		[n = 8447]	
<10 km	1.00		1.00	
≥10 km	1.17(0.96–1.43)		1.17*(1.01–1.36)	
Distance from clinic (Km)	[n = 4138]		[n = 8447]	
0–2.49	1.00	1.00	1.00	
2.5–4.99	1.65***(1.23–2.21)	1.54**(1.12–2.16)	1.00(0.84–1.21)	
5–9.99	1.36*(1.06–1.75)	1.25(0.94–1.66)	1.01(0.87–1.22)	
10–49.99	1.57**(1.20–2.15)	1.39(0.99–1.94)	1.19(0.95–1.47)	
50–100	2.22(0.76–6.54)	1.65(0.54–5.04)	1.33(0.44–3.98)	
>100	2.19(0.64–7.59)	2.31(0.63–8.57)	1.14(0.48–2.66)	

AOR – adjusted odds ratio from multivariate models adjusted for all other included factors.

* p<0.05;

** p<0.01,

*** p<0.001. 95% CI presented in parenthesis.

Þ Through June 30, 2011;

## Discussion

Overall, 18.8% of LH and 22.4% of MPC patients informed of their ART eligibility still failed to start ART, similar to recent studies from Northern [26] and Southern [11] Malawi. Among demographic factors, the odds of starting ART were lowest among patients ages 15–24 and highest among patients older than 35 years. TB co-infection at eligibility, a WHO stage 3 condition, increased the likelihood of starting ART at both clinics. In multivariate models, patients eligible for ART based on WHO stage 3 or 4 were much more likely to start than patients with WHO stages 1–2 combined with CD4 criteria at both clinics. Increasing months in pre-ART dramatically increased a patient’s likelihood of ART initiation: patients with 1–12 months in the pre-ART period were at least 11 times more likely to start ART than their peers with less than a month in pre-ART. Lastly, distance from clinic LH increased the likelihood of starting ART at LH for some patients, but distance was not a significant influence at MPC. Previous research helps illuminate the findings.

First, we found pre-ART care, the time between ART registration and determination of ART eligibility, is the most significant predictor of starting ART among eligible patients. Patients who were immediately eligible at initial registration had lower odds of starting ART at both LH and MPC. However, patients who spent between 1–3 months in the pre-ART period were more than 28 times at LH and 49 times at MPC more likely to start ART, an effect that decreased slightly, but remained highly significant, even after more than 12 months in that transitional time. The larger effect of time in pre-ART at MPC over LH may be due to the location of MPC near a large hospital and transit center. Although MPC may see more patients due to easier access to services, more of those patients may be mobile and less prepared to start treatment thus increasing the effect of additional pre-ART time. We acknowledge that some eligible patients may have started ART elsewhere [13], [27], [28], died before they could initiate ART [9], [26], [27], [29], [30], or simply not wanted to start ART [31]. However, our finding that pre-ART time increases the likelihood of ART initiation is consistent with regional findings on the individual- and service-level factors that influence patient retention during this period. Studies from Uganda [32] and Malawi [33] suggest that ART uptake is influenced by patient acceptance of their HIV-positive status, disclosure, and preparation for ART initiation. Among healthcare factors, patients from a South African clinic who returned for one or more routine, pre-ART visits had multiple exposures to care providers who appeared to increase patient motivation and refute prevalent myths about ART, ultimately reducing delays in ART initiation [34]. A study from Kenya found that provision of other pre-ART services, including bi-annual CD4 testing and free prophylaxis therapy, reduced pre-ART attrition [35].

Second, as other studies from the region [13] and Malawi [26] note, healthier patients may not perceive the need to start ART. Both at LH and at MPC, patients who were younger, had higher BMI, or eligibility based on CD4, possibly reflecting better health, were less likely to start ART than those who were older, with lower BMI or eligible by WHO stages 3 or 4. Among younger and healthier patients, perceptions of health and wellness may combine with lack of preparation to start lifelong ART, adding additional barriers to ART initiation. Also, in contrast to studies that found increased barriers to ART initiation among TB co-infected patients [4], [36], including in our own clinic [37], we found that TB co-infection at eligibility increased the odds of starting ART. Although both LH and MPC provide TB treatment, a stronger, positive effect of TB co-infection at eligibility on ART initiation was evident at MPC, a purposefully integrated clinic that also serves as a national TB diagnosis, treatment, and follow-up facility. Possibly, sicker patients with easy access to comprehensive TB/ART care may be more likely to initiate ART, suggesting an additional benefit of service integration.

Furthermore, although socio-economic factors likely contribute to attrition among pre-ART patients, the two socio-economic variables available in our routine data, distance and employment status, were less influential than anticipated. Previous research from public clinics in the region noted transportation costs as a barrier to returning for subsequent visits [13], [26], [30], [32], [38], [39], [40]. However, findings from this study suggest that employment and distance to clinic were not significant deterrents to ART initiation. Several factors may make our clinic cohorts different. First, LH was the first public clinic providing ART and serves as a center of excellence for the region; patients who started there may continue to seek care there despite transportation costs or distances. At MPC, distance may play a lesser role because of its heightened accessibly next to the bus station in the central business district. Moreover, lingering stigma, perceived or experienced, may still motivate patients to seek care far from their neighborhoods and nearby clinics. Lastly, it is possible that patients who took initiative or had resources to seek care from more distant places were also those more driven to continue care and initiate treatment.

These findings should be considered within the following limitations. First, patients who were eligible earlier in the study period had more time to start ART; some patients who became eligible closer to July 1, 2011 may have started ART after the study censure date of September 30, 2011. However, almost 70% of LH and MPC patients start ART within 2 weeks of eligibility, with median days between eligibility and ART start of 19 days and 21 days at LH and MPC, respectively, reducing the effect of this potential bias. Second, this study used only routine service statistics; other social, economic, or structural data was not available. Third, pre-ART data over multiple visits is not stored. Therefore, the number and type of visits between initial registration and eligibility cannot be determined, decreasing our ability to determine what service factors and visit frequency may be influential during that period. Fourth, patients who left pre-ART care before they were informed of their eligibility, a factor more likely to affect those who needed to return for at least one additional visit to receive CD4 results, were not included. Program attrition among patients who drop out of care earlier, including those not informed of their eligibility, merits further attention. Lastly, several groups of patients were excluded from this analysis (Table 1), including children, pregnant women, and those with incomplete data such as transfer-in patients, decreasing the sample size and reducing generalizability to other populations or clinics. Analysis of eligible patients who were excluded compared to those included [not shown] indicated that excluded patients at both LH and MPC were significantly different in sex, age, eligibility criteria, time in pre-ART, and starting ART; therefore, caution must be used in generalizing these results especially to transfer patients who comprise 68% and 54% of excluded patients at LH and MPC, respectively. Despite these limitations, LH and MPC are large, well-established, urban providers of public services, and information on the drivers and deterrents of program attrition among identified, ART- eligible patients is likely representative of similar groups of eligible, urban patients in Malawi.

### Conclusion

The findings suggest several steps to reduce program attrition during this period at both Lighthouse clinics and in Malawi. First, as those with more time between registration and eligibility appear more likely to start. Therefore, testing and referral process that encourage early entry into pre-ART care coupled with improved counseling on both ART preparedness and transiting to lifelong treatment may decrease attrition. Second, pre-ART counseling guidelines should be revisited to resonate with those who are eligible by CD4 and younger patients, many of whom may not feel sick. Third, those eligible by CD4 appear less likely to start due, in part, because they have to return to be informed of eligibility. Implementation of point of care CD4 testing as tested in neighboring countries may prove beneficial to reduce ART initiation delays [41], [42]. Lastly, although LH and MPC have a successful client follow-up program for patients on ART [43], this program should be extended to include pre-ART patients in this clinic setting and throughout Malawi, where possible. Adoption of one or more of these recommendations may help reduce systematic weaknesses in the continuum of HIV-related services, move more eligible patients swiftly onto ART, and improve patient health and outcomes over time.
